# A 12 week, randomized, double-blind, placebo-controlled clinical trial for the evaluation of the efficacy and safety of HT083 on mild osteoarthritis

**DOI:** 10.1097/MD.0000000000020907

**Published:** 2020-07-10

**Authors:** Donghun Lee, Seok Jung Kim, Hocheol Kim

**Affiliations:** a Department of Herbal Pharmacology, College of Korean Medicine, Gachon University, Seongnam; b Department of Orthopedic Surgery, College of Medicine, The Catholic University of Korea; c Department of Herbal Pharmacology, College of Korean Medicine, Kyung Hee University, Seoul, Republic of Korea.

**Keywords:** *Commiphora myrrha*, clinical trial, HT083, osteoarthritis, *Paeonia lactiflora*, pain

## Abstract

**Background:**

The increasing prevalence of osteoarthritis among the old population worldwide is a great concern. Two of the biggest complaints of OA patients are joint pain and inflammation. Currently, people are relying on non-steroidal anti-inflammatory drugs (NSAIDs) and steroids to control pain and inflammation. However, long-term use of these pharmaceutical drugs has negative health consequences in the elderly, including gastro-intestinal, respiratory, and renal diseases. Natural products are receiving more attention than ever as alternative treatments against OA for their efficacies and safety. The root of *Paeonia lactiflora* Pal and the gum resin of *Commiphora myrrha* have been used as analgesics and anti-inflammatory agents since ancient time. A new herbal formula composed of *P. lactiflora* root and *C. myrrha* gum resin extracts, known as HT083, has shown promising antinociceptive and anti-inflammatory effects in a rodent model of OA. We design this study to investigate the safety and the efficacy of HT083 to prevent OA in patients with mild OA.

**Methods:**

This is a randomized, double-blind, and placebo-controlled study. A total of 100 eligible participants will be divided into two groups and will be given HT083 and a placebo for 12 weeks in 1:1 ratio. Treatment results will be assessed using a visual analog scale (VAS), Korean-Short Form health survey-36 score (SF-36), personal evaluation, and laboratory analysis.

**Discussion:**

This trial is expected to provide clinical evidence on the effectiveness and the safety of HT083 as a natural treatment for mild OA.

**Trial registration:**

Korean Clinical Research Information Service (CRIS) number KCT0004925 Registered on 2020.04.16.

## 1 Introduction

Osteoarthritis (OA) is known as a degenerative joint disease that affects millions of elderly people throughout the world. In most cases, OA patients experience synovial inflammation and cartilage loss, which causes pain and immobility.^[1]^ Nearly 50% of the people aged 65 or older on a global scale are believed to have OA symptoms.^[2]^ The pharmacological drugs such as non-steroidal anti-inflammatory drugs (NSAIDs) and steroids that are currently being used to manage OA pain and inflammation have adverse health effects if used for a long time. As a consequence, natural medicines, for their efficacy and safety, are being prioritized as a treatment for OA.

*Paeonia lactiflora* Pall root and *Commiphora myrrha* (Nees) Engl. gum resin are known for their pain-relieving and anti-inflammatory properties.^[3,4]^ The application of these herbs as traditional medicines dates back to ancient time. The use of herbal formula in Chinese medicine has been practiced for centuries for better functionality and safety than the individual herbs.^[5]^ An herbal formula composed of *P. lactiflora* and *C. myrrha* extracts named as HT083 has shown potent antinociceptive, anti-inflammatory, and cartilage protective activities in an animal model of OA in our previous study. We hypothesize that HT083 will prevent OA pain and inflammation in human. This placebo-controlled double-blind study will evaluate the efficacy and safety of HT083 in human subjects with mild OA.

## 2 Methods and analysis

### 2.1 Study design

**Figure 1:**
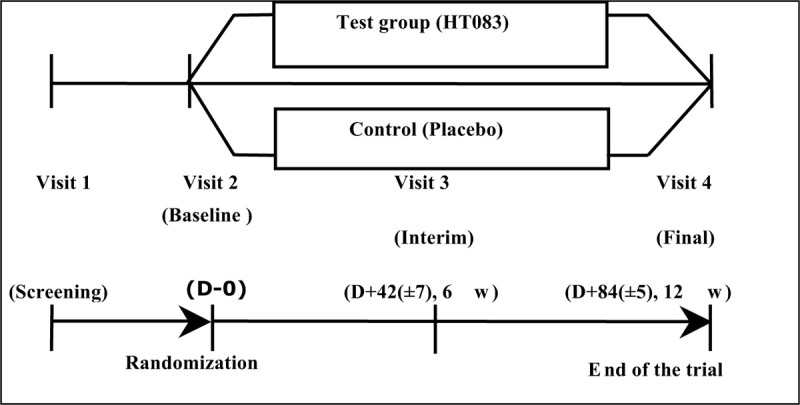
Flow diagram of the trial.

This is a randomized, 12-week, double-blind, placebo-controlled study. The current protocol is registered at Neonutra Co. Ltd. (protocol number NM_HT083hn, version 1.1) on 14 Oct 2019 sponsored by Neumed Co., Ltd. The participants will be recruited at Uijeongbu St. Mary's Hospital, The Catholic University of Korea. After obtaining a written consent from all the participants, the qualified subjects will be randomly divided into HT083 and placebo groups. The treatment will continue for 12 weeks, and during that period, the participants will be provided necessary guidance on maintaining daily diets and activities. The subjects will be required to visit the hospital four times throughout the study for assessment. The assessments will be scheduled as screening (week −2 to 0), baseline (week 0), and interim (at the end of week 6) and the final (at the end of week12). The flow chart of the trial is shown in Figure 1.

### 2.2 Ethics approval and consent of the participants

The trial protocol (NM_HT083, version 1.0, date October 14, 2019) was approved by the ethics committee of the Uijeongbu St. Mary's Hospital, The Catholic University of Korea [UC19HDSE0143]. The participants will be informed about the study purpose, procedure, inclusion and exclusion criteria, test product, and the possible side effects. A written consent will be obtained from all the participants and each participant will be given the signed consent form by the investigator. All the details of the study and the personal information of the participants will be kept confidential and only be accessible to the authorized investigators. The final trial dataset will be accessible to the principal investigator and the sponsor. The trial protocol and the dataset can be accessed from the corresponding author upon a legitimate request. After completion, the results of the trial will be published in a peer-reviewed journal. The investigators have no financial or any other interests in regards to the trial.

### 2.3 Participants

#### 2.3.1 Inclusion criteria

Subjects who meet all the below criteria will be qualified and enrolled in the study:

- Male or female adults aged between 35 and 70 years of age.
- Subjects with a visual analog scale (VAS) score of more than 30 mm
- Subjects with Kellgren and Lawrence grade I or II determined by X-ray
- Subjects who agree to participate in the clinical trial voluntarily and sign the informed consent form.

#### 2.3.2 Exclusion criteria

Subjects who have one or more of the following characteristics will not be considered for the study.

- A person who has arthritis other than OA.
- Those who have clinically significant cardiovascular, autoimmune, infectious or neoplastic diseases in addition to joint pain.
- Hypertension patients not controlled by drugs with more than 160 mmHg systolic blood pressure or more than 100 mmHg diastolic blood pressure.
- Diabetic patients whose fasting blood glucose level is above 180 mg/dl).
- A person who has aspertate aminotransferase (GOT) or alanin aminotransferase (GPT) level 3 times higher than the normal upper limit.
- Abnormal creatinine level (more than twice the normal upper limit).
- Those who have taken arthritis medicines or dietary supplements for joint health within 2 weeks of visit.
- Subjects who have taken physical or herbal therapies (acupuncture, depression, moxibustion, etc.) for degenerative bone disease within 2 weeks of visit.
- Subjects who participate in other intervention trials within two months of visit 1 or plan to participate in other intervention trials after the commencement of this study.
- Women who are pregnant, lactating, or planning to become pregnant within 3 months.
- A person with mental illness or drug addiction or who is under anti-depression treatment.
- A person who is sensitive or allergic to food ingredients.
- A person who has trouble or difficulties participating in the trial as judged by the investigator.

#### 2.3.3 Withdrawal and dropout

According to the Declaration of Helsinki, any participant has the right to leave the trial at any time without giving a reason. Participants might be withdrawn from the study to ensure their safety by the investigator. Investigators also possess the right to withdraw a participant from the study for any of the following reasons:

- A significant violation of the protocol.
- In case of a serious adverse event.
- Subject's refusal to continue with the trial.
- Subject withdraws the consent.
- When a subject is unable to ingest the test product or the placebo.
- Receiving any medication or treatment which may interfere or influence the test result.
- For any safety reasons.
- Pregnancy.

### 2.4 Recruitment

The participants will be recruited under Uijeongbu St. Mary's Hospital, The Catholic University of Korea, South Korea. The recruitment for the clinical trial will be advertised through posters in public places like hospitals and subways after the approval from the institutional review board. The posters will contain information on the aim and the procedure of the trial including the sponsor, the hospital, selection criteria, and the product details.

### 2.5 Randomization and blinding

A randomization list will be created by an independent analyst using block randomization method in SAS version 9.2 (USA). Participants will be given a randomized code and will receive the treatment product labeled with the same code. Participants will be randomly allocated to either the test or the placebo group in a 1:1 ratio. The placebo will be used to compare the efficacy of the test product. All participants and the research personnel will remain blinded to the assigned treatment until the end of the trial. The appearance of the test food and the placebo product will be identical. Unblinding will be permissible under emergency circumstances which are defined in the study protocol.

### 2.6 Intervention

The test product is a 500 mg white tablet containing HT083, crystalline cellulose, silicon dioxide, magnesium stearate, hydroxypropylmethylcellulose, glycerine fatty acid ester, and titanium dioxide. A 500 mg placebo product contains crystalline cellulose, maltodextrin, silicon dioxide, magnesium stearate, hydroxypropylmethylcellulose, fatty acid ester, and titanium dioxide. Participants will be instructed to take 2 tablets a day, 1 in the morning and 1 at night in order to have a dose of 1 g per day for 12 weeks. The unused tablets should be required to be returned to the test center for evaluating the subjects’ adherence to the trial. The test and placebo products have been manufactured by NeoNutra Co. Ltd (Seoul, Korea) in a GMP-certified facility. The trial schedule is presented in Table 1.

**Table 1:**
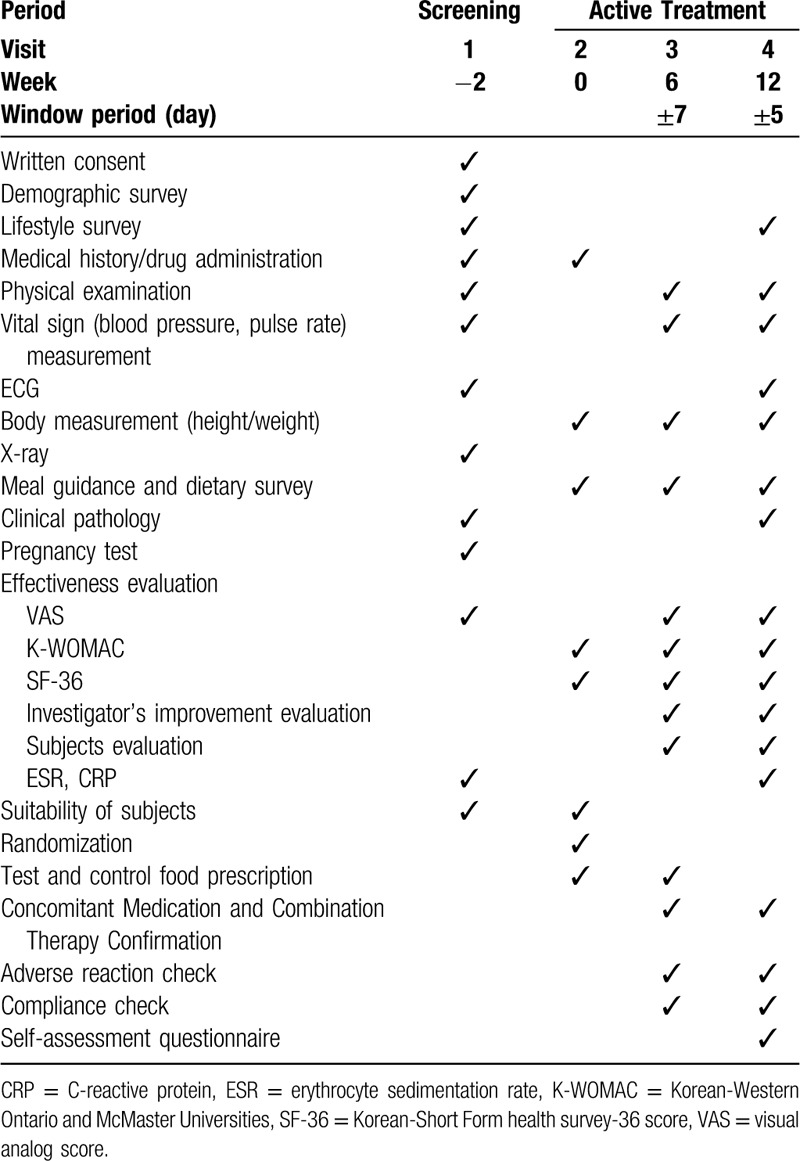
Trial Schedule.

### 2.7 Prohibited concomitant drugs and therapies

The use of the following drugs and therapies may interfere with the evaluation of safety, efficacy, and tolerability of the test product. Therefore, the drugs and therapies listed below will be restricted during the intervention period:

- Acetaminophen.
- Aspirin and anti-inflammatory drugs.
- Medication for degenerative arthritis.
- Hyaluronic acid and corticosteroids.
- Korean traditional medicines to treat OA.
- Dietary supplements for joint health.
- Physiotherapy or herbal treatments for degenerative arthritis (acupuncture, cupping therapies, moxibustion).

All Information about concomitant drugs and therapies will be recorded in the case report form.

### 2.8 Outcome evaluation

The following outcomes will be evaluated by trained evaluators during each visit.

#### 2.8.1 Primary outcomes

- Korean-Western Ontario and McMaster Universities (K-WOMAC) index. The K-WOMAC index will measure changes in joint pain at the end of week 6 and 12 to compare those with the baseline scores.

#### 2.8.2 Secondary outcomes

- Visual analog scale (VAS), which evaluates joint pain as compared with the baseline score.
- Korean-Short Form Health Survey 36 (KSF-36). The KSF-36 index will assess the physical and mental states of the subjects at the end of week 6 and 12.
- Subject's self-assessment on the change of physical and mental conditions.
- Investigator's evaluation based on subject's physical activity, symptoms, and emotional state.
- Erythrocyte sedimentation rate (ESR) and C-reactive protein (CRP) levels to measure the inflammatory markers after 12 weeks of study.

#### 2.8.3 Safety outcomes

The safety outcome variables are adverse event, vital signs (blood pressure, pulse rate, body weight etc.), clinical test results, and echocardiogram result.

### 2.9 Data management and quality control

All data gathered throughout the study will be recorded in the case report form in a timely manner and will be open for the monitor to review the status of the participants. The Clinical Research Associate from the sponsor will regularly monitor and review the data with the investigator for any missing or spurious data. All the resolutions regarding the data will be recorded in the database. Clean dataset will be provided to the analysts. A data monitoring committee (DMC) will not be needed as the test product is low-risk and there will be no interim analysis. Auditing will not be conducted for this trial.

### 2.10 Sample size

The sample size will be calculated following a previously described method where the VAS score in the test group decreased from ∼4.2 to ∼25.4, whereas the VAS score in the placebo group increased from ∼40.9 to ∼41.^[6]^ We also assume that the change in the VAS score in our test and placebo groups will be −16.6 and +0.1, respectively. The following parameters will be considered to determine the size of the sample:

Superiority test

Level of significance, a = 0.05 Two-sided test

b = 0.2, power of test = 80%

Ratio of the number of subjects in the test and placebo groups, l = 1, n_t_ (subject number of the test group) = ln_c_ (subject number of the placebo group)

Difference between the groups, D = 16.7

Standard deviation, s = 27.04 (in both groups)

We assume that with a dropout rate of 25%, 37 participants will be included in each group.

### 2.11 Statistical analysis

All statistical analyses will be performed using SAS version 9.2 (SAS institute Inc., USA). The total score of K-WOMAC and the change of score of each item will be analyzed using paired *t* test, and the degree of change between test and the control group at each time point will be determined according to normality satisfaction. A *t* test or Wilcoxon rank sum test will be performed to evaluate whether there is a statistically significant difference. In addition, a generalized linear model (GLM) will be performed with the following variables (age, gender, obesity). The total scores of VAS, SF-36, ESR, and CRP and the change of scores of each item will be analyzed by paired *t* test, and the degree of change between the test group and the control group at each time point will be normalized. Two sample *t* test or Wilcoxon rank sum test will be used to evaluate whether there is a statistically significant difference. In addition, the GLM will be implemented with the following variables (age, gender, obesity) as the covariate, and if there is a statistically significant difference among the baseline characteristics, the GLM will be considered as the covariate. All treatment-emergent adverse events (TEAEs) that occur after ingestion of human trial foods will be coded according to MedDRA, and all adverse events that will occur after ingestion of human trial foods will be tabulated and evaluated for incidence. The proportion of human subjects with adverse reactions between groups will be calculated and analyzed using Chi-square test or Fisher exact test.

## 3 Discussion

As a leading cause of immobility among aging population throughout the world, OA is getting increasing attention. It is a chronic inflammatory joint disease that degrades the joint cartilage and alters the subchondral bone causing unbearable pain, stiffness, and even the loss of movement.^[7]^ Current first-line therapies consisting of NSAIDs and COX-2 inhibitors have limited success and are associated with negative health consequences. In this context, natural products are increasingly being in the focus of research in developing a new generation OA treatment, which would be efficient and risk free at the same time.

*P. lactiflora* is a Chinese folk medicine that have been used to treat a number illnesses, including pain, inflammation, and autoimmune diseases for over a millennia.^[8]^ The root of *P. lactiflora* contains several bioactive compounds, such as paeoniflorin, albiflorin, penta-O-galloyl-β-d-glucose with anti-inflammatory and other health functions.^[9]^ A recent study reported that paeoniflorin, obtained from *P. lactiflora* can mitigate inflammatory pain by suppressing Akt-NF-kB pathway.^[10]^*C. myrrha*, a native plant to Northeastern Africa, has been used to treat rheumatoid arthritis, sinusitis, and other illnesses.^[11]^ Both *C. myrrha* and *P. lactiflora* have been historically used as natural antinociceptives since ancient times, and both have proven functions against peripheral and central pains.^[12,13]^ In traditional medicine, herbal formulas are regarded as superior to individual herbal treatments in terms of potency and safety, as individual herbs can produce synergistic effects and neutralize any potential toxicity through interactions.^[14]^ We combined *P. lactiflora* and *C. myrrha* extracts in a 3:1 ratio, as the most effective ratio based on our preliminary data and termed as HT083. This new herbal formula has shown analgesic, anti-inflammatory, and cartilage-protective functions in a rodent model of OA. We hypothesized that HT083 will have similar anti-osteoarthritic effects in human.

This study has few limitations. First, this trial will be conducted on only one ethnic group. Therefore, it is unknown whether our data will be representative of other parts of the world other than Korea. Second, the small size of the test group makes it hard to generalize the data. However, given the analgesic and anti-inflammatory properties of *P. lactiflora* and *C. myrrha* as well the in vivo anti-osteoarthritic effects of HT083, we hope that this trial will demonstrate the efficacy and safety of HT083 in patients with mild OA.

## Author contributions

**Conceptualization:** Hocheol Kim

**Methodology:** Seok Jung Kim, Donghun Lee

**Project administration:** Seok Jung Kim

**Supervision:** Seok Jung Kim

**Writing – original draft:** Donghun Lee

**Writing – review & editing:** Seok Jung Kim, Hocheol Kim.
